# Novel ENU-Induced Mutation in *Tbx6* Causes Dominant Spondylocostal Dysostosis-Like Vertebral Malformations in the Rat

**DOI:** 10.1371/journal.pone.0130231

**Published:** 2015-06-19

**Authors:** Koichiro Abe, Nobuhiko Takamatsu, Kumiko Ishikawa, Toshiko Tsurumi, Sho Tanimoto, Yukina Sakurai, Thomas Lisse, Kenji Imai, Tadao Serikawa, Tomoji Mashimo

**Affiliations:** 1 Department of Molecular Life Science, Tokai University School of Medicine, Isehara, Kanagawa, Japan; 2 Department of Biosciences, School of Science, Kitasato University, Sagamihara, Kanagawa, Japan; 3 Institute of Laboratory Animals, Graduate School of Medicine, Kyoto University, Kyoto, Japan; 4 MDI Biological Laboratory, Davis Center for Regenerative Biology and Medicine, Bar Harbor, Maine, United States of America; Institute of Molecular and Cell Biology, SINGAPORE

## Abstract

Congenital vertebral malformations caused by embryonic segmentation defects are relatively common in humans and domestic animals. Although reverse genetics approaches in mice have provided information on the molecular mechanisms of embryonic somite segmentation, hypothesis-driven approaches cannot adequately reflect human dysmorphology within the population. In a *N*-ethyl-*N*-nitrosourea (ENU) mutagenesis project in Kyoto, the *Oune* mutant rat strain was isolated due to a short and kinked caudal vertebra phenotype. Skeletal staining of heterozygous rats showed partial loss of the cervical vertebrae as well as hemivertebrae and fused vertebral blocks in lumbar and sacral vertebrae. In homozygous embryos, severe displacement of the whole vertebrae was observed. The *Oune* locus was genetically mapped to rat chromosome 1 using 202 backcross animals and 50 genome-wide microsatellite markers. Subsequently, a miss-sense mutation in the *Tbx6* gene was identified in the critical region. Although the mutation is located within the T-box domain near a predicted dimmer-interface, *in vitro* experiments revealed that the *Tbx6* variant retains normal DNA binding ability and translational efficiency. However, the variant has decreased transcriptional activation potential in response to Notch-mediated signaling. Recently, it was reported that a dominant type of familial spondylocostal dysostosis is caused by a stoploss mutation in *TBX6*. Thus, we propose that partial dysfunction of *Tbx6* leads to similar congenital vertebral malformations in both humans and rats. The *Oune* strain could be a unique animal model for dominant spondylocostal dysostosis and is useful for molecular dissection of the pathology of congenital vertebral malformations in humans.

## Introduction

The vertebral column provides structural strength and flexibility to the body. It is derived from somites, the bilateral segmented structures in the embryo [1]. With axial elongation of the developing embryo, the unsegmented paraxial mesoderm called, presomitic mesoderm (PSM), plays a key role for somitogenesis [2]. The most anterior part of PSM is segmented one-by-one as newly formed somites. Thus, defects in somitogenesis cause severe congenital vertebral malformations [3]. In the mouse, many spontaneous mutant lines with vertebral malformations have been collected and intensively analyzed [4]. By forward genetics approaches, many of causative genes for the skeletal phenotypes have been identified. In addition, knockout mouse lines with defects in somitogenesis have been generated by genetic engineering. This increased knowledge of the molecular mechanisms of vertebral segmentation contributes to the positional candidate cloning of causative genes for familial vertebral malformations in humans [5].

Spondylocostal dysostosis is characterized by multiple vertebral segmentation defects; patients exhibit short trunk dwarfism with nonprogressive mild scoliosis [6]. The skeletal anomalies include rib fusion and/or deletion called crab-like thorax as judged by radiographic diagnosis. By familial analyses, both dominant and recessive types of spondylocostal dysostosis have been identified. Autozygosity mapping of consanguineous families [7] in combination with candidate sequencing revealed four recessive mutations in the Notch signaling pathway (*DLL3*, *MESP2*, *LFNG*, *HES7*) [5]. In contrast, dominant types of spondylocostal dysostosis are relatively rare [8]. But recently the first dominant mutation of spondylocostal dysostosis was reported [9]. In this family, the stop codon of the *TBX6* gene was mutated. Accordingly, the variant TBX6 with additional 81amino acids to the carboxyl terminus resulted in the attenuation of transcriptional activation *in vitro*.

The T-box family members have a common DNA binding domain [10]. The first T-box gene was discovered by positional cloning of the *Brachyury* (or *T*, for short-tail) mutations in mice [11]. Thereafter, genes having homology to the *Brachyury* DNA binding domain were identified and defined as T-box genes [12]. T-box genes are important developmental regulators of a wide range of tissues and organs, as well as major contributors to several human syndromes [10]. Spontaneous and induced mutations of *Tbx6* in mice have been well characterized [13–16]. Although those mutations cause developmental and morphological abnormalities in homozygotes, almost no skeletal phenotypes were detected in heterozygotes [9]. This differs from human patients where vertebral segmentation defects are observed in heterozygotes. Here, we present a novel *N*-ethyl-*N*-nitrosourea (ENU)-induced semidominant mutation, *Oune* (for tail curvature in Japanese), in rats. *Oune/+* rats show malformations in the entire vertebral column, and positional candidate cloning identified a missense mutation in the *Tbx6* gene. Thus *Oune* could be a novel animal model for dominant spondylocostal dysostosis.

## Materials and Methods

### Rats

The first *Oune* rat was identified in a gene-driven ENU mutagenesis project in Kyoto University (details are descried in Mashimo *et al*.) [17]. Briefly, we administrated two intraperitoneal injections of 40 mg/kg ENU at a weekly interval to 9- and 10- week-old F344/NSlc (F344) males (Japan SLC). ENU-treated males were mated with F344 females to generate G1 offspring. The *Oune* rat was backcrossed more than ten generations on the F344/NSlc inbred background to eliminate mutations potentially induced by ENU mutagenesis elsewhere in the genome (mean mutation frequency was approximately 1 in 4×10^6^ bp). The *Oune* rat has been deposited into the National Bio Resource Project Rat in Japan (NBPR-Rat No. 0464) and is available from the Project (http://www.anim.med.kyoto-u.ac.jp/nbr). Animal care and experimental procedures that were used were approved by the Animal Research Committee, Kyoto University and were carried out according to the Regulation on Animal Experimentation at Kyoto University.

### Genetic mapping and candidate sequencing

A total of 202 N2 rats were produced from a (F344-*Oune/+* × BN/SsNSlc)F1 × BN/SsNSlc backcross. Genomic DNA was prepared from tail biopsies using an automatic DNA purification system (PI-200; Kurabo, Osaka, Japan). For genetic mapping of the *Oune* locus to a specific chromosomal region, genomic DNA samples from *Oune/+* N2 rats (n = 15) judged by a kinked tail and from +/+ rats (n = 15) judged by a normal tail were pooled and used for genotyping PCR. We performed genome-wide scanning on the pooled DNA samples using a panel of 50 simple sequence length polymorphism (SSLP) markers that cover all the autosomal chromosomes (S1 Table). All PCRs were performed for 30 cycles (denaturation at 94°C for 30 s, annealing at 60°C for 1 min, and extension at 72°C for 45 s), using Taq DNA polymerase (Takara Bio). PCR products were examined on 4% agarose gels with ethidium bromide staining. For fine mapping within the *Oune* region, nine SSLP markers on chromosome 1 were added (S2 Table). The genotypes for the *Oune* locus in the 202 N2 rats were identified on the basis of tail phenotype at three to four weeks of age. For DNA sequencing of *Tbx6*, we used primers covering exonic regions (S3 Table) for PCR amplification, and the PCR products were sequenced by standard Sanger sequencing using the PCR primers.

### Skeletal analysis and *in situ* hybridization

Skeletal preparations of newborn rats were generated as described previously [18]. *In situ* hybridization on histological sections was performed as described previously [19,20]. The mouse *Pax1* [21], *Uncx4*.*1* [22], and *Dll1* [23] RNA probes were used for *in situ* hybridization on sections of rat embryos. For whole mount *in situ* hybridization, rat cDNA were cloned into pCR4-TOPO (Invitrogen) using the following oligonucleotides: 5'-GGTGGGGATATTCGAGATT and 5'-AACAAATTGGCGTGGCTTAC (*Tbx6)*; 5'-TGGATCCTCCTTTCCCAGATG and 5'-GGTGGATTGGCAGACTTGTT (*Mesp2)*; 5'-GGACCTCTGGCGTATTTGAG and 5'-CTCACAGTTGGCCCCTGTAT (*Dll1)*. The insert of the plasmids were sequenced and used for synthesis of RNA probes.

### Expression constructs

Rat *Tbx6* (*rTbx6*) sequences (Genbank Accession No NM_001108920.1) including the coding region were PCR cloned using the following oligonucleotides: Ins-Oune-F, 5’-CACCATGTACCATCCACGAG and Tbx6_ISH-R, 5’-AACAAATTGGCGTGGCTTAC. For mouse *Tbx6* (*mTbx6*, Genbank Accession No NM_011538.2), the following oligonucleotides were used: mTbx6-fullCDS1-L, 5’-ATGTACCATCCACGAGAGTTGTA and mTbx6-fullCDS1-R, 5'-ATCAAGGGAAGATGGCTATGG. The RT-PCR fragments of *rTbx6* and *mTbx6* were cloned into pcDNA/V5/GW/D-TOPO (Invitrogen) and pBluescript KS+ (Agilent), respectively. The *Oune* mutation corresponding to mouse *Tbx6* was introduced by PCR-based site-directed mutagenesis using PrimeSTAR Mutagenesis Basal Kit (Takara Bio) according to manufacture’s protocol using two oligonucleotides: 5’-GTCTACAATCACCCTGACTCTCCT and 5’-AGGGTGATTGTAGACACGGTCGGG. The desired mutation was confirmed by Sanger sequencing.

### Western blotting

293T cultured cells were grown in Dulbecco modified Eagle medium (DMEM) with 10% fetal bovine serum. The cells were plated at 3×10^5^ cells per 35-mm dish, and after 24 hours were and transfected with pcDNA3 or pcDNA3/S-tag-mTbx6 using TransIT-LT1 (Mirus) [24]. After 24 hours, cell lysates were prepared using RIPA buffer, and subjected to sodium dodecyl sulfate (SDS)-10% polyacrylamide gel electrophoresis (PAGE) and subsequent immunoblotting. The anti-S-tag antibody was purchased from Novagen, and the anti-USF2 antibody was purchased from Santa Cruz Biotechnology.

### *In vitro* transcription/translation

Proteins were synthesized from corresponding plasmids using TNT T7 Quick Coupled Transcription/Translation System (Promega) in the presence of [^35^S]-methionine, and analyzed by SDS-PAGE.

### Electrophoretic mobility shift assay (EMSA)

Tbx6 proteins were synthesized using an *in vitro* transcription/translation system (Promega). The following oligonucleotides were annealed with the complementary oligonucleotides and used as probes: Tbind, 5’-GGCTAGTCACACCTAGGTGTGAAATT-3’; Tbind-half, 5’-ATCGAATTCAGGTGTGAAATGGATCCACT-3’. The oligonucleotide sequence information was from White and Chapman [25], except adding 2xG at the 5' end for klenow fragment labeling with [α-^32^P]-dCTP in Tbind. EMSA was carried out as described previously [24].

### DNA transfection and luciferase assay

C2C12 cells were plated at 5×10^4^ cells per 15-mm dish and cultured in DMEM with 10% fetal bovine serum. After 24 hours they were transfected with 200 ng of a firefly luciferase promoter-reporter plasmid, P2Ewt [26], 2.5 ng of a Renilla luciferase internal control plasmid, pRL-SV40 (Promega), and 50 ng of the rat wild type Tbx6 (rTbx6 wt) or *Tbx6* ^*Oune*^ (rTbx6 mut) expression construct described above, in the absence or presence of 2.5 ng of the NICD expression construct [27], using TransIT-LT1 (Mirus). In experiments with a mixture of Tbx6 expression constructs, the experimental conditions are same except the amount of transfectants; 100 ng of rTbx6 wt, 100 ng of rTbx6 mut, and a mixture of 50 ng of rTbx6 wt and 50 ng of rTbx6 mut were used instead. Luciferase activity was measured 24 hours after transfection using the Dual-Luciferase Reporter Assay System (Promega).

## Results

**Fig 1:**
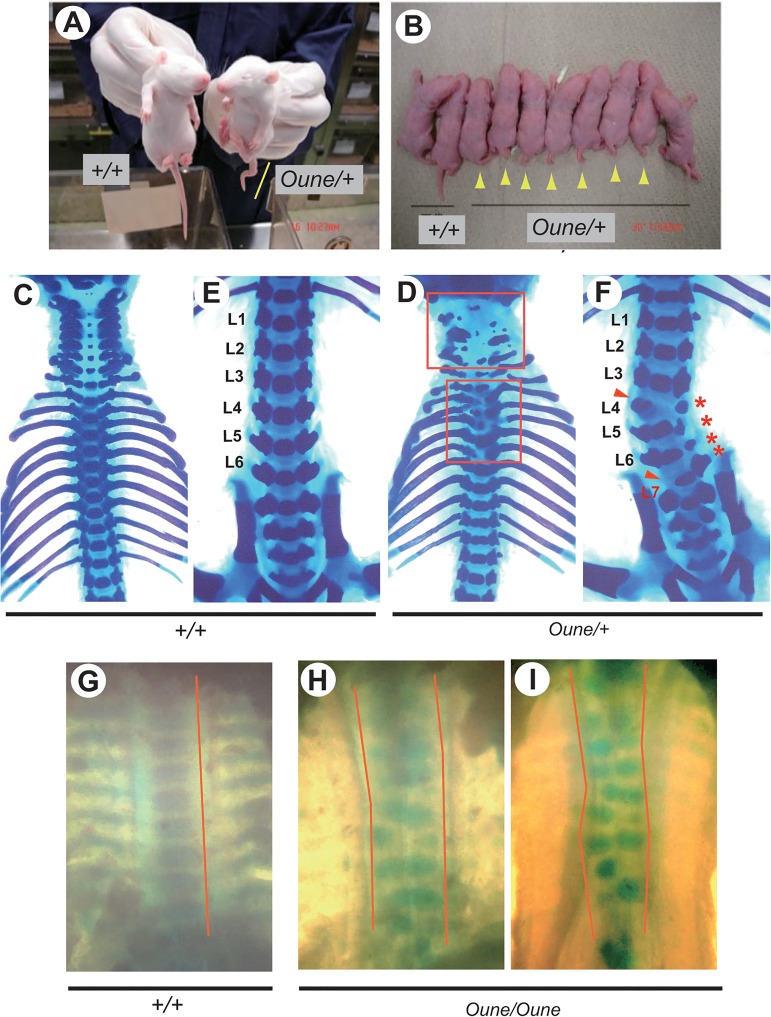
Morphological abnormalities of vertebral column in ENU-induced *Oune* rats. (A, B) Perinatal and newborn offspring derived from (*Oune/+* x *+/+*) mating pairs. *Oune* rats were distinguished from their siblings because of kinky tails (yellow bar and arrowheads). (C-F) Ventral view of axial skeletons of newborn wild type and *Oune/+* siblings. In the cervical and thoracic region, *Oune/+* rats showed loss and malformations of vertebrae (D, boxes). In the lumbar and sacral region of *Oune/+* animals, vertebrae were malformed and laterally bent (F, asterisks). An extra lumbar vertebra was frequently observed (F, L7). (G-J) Ventral view of axial skeletons of E15.5 *Oune* siblings. Wild type embryos showed ordered thoracic vertebral blocks along the anterior-posterior axis (G, bars). In *Oune/Oune* embryos, vertebral blocks were located along two different axes (H, I bars) with loss of rib formation. Original magnification: 12.5x (C, D); 10x (E, F); 32x (G-I).

The first *Oune* rat was distinguished from G1 offspring due to a short and kinked tail (Fig 1A). From mating pairs between *Oune* and wild type F344 animals, offspring showed a mix of normal and congenitally kinked tails (Fig 1B). By repeated backcrossing with the F344 wild type strain, *Oune* was established and maintained as a dominant mutant strain on the F344 genetic background. Skeletal analysis of *Oune/+* newborn animals showed complete loss of atlas (C1) and axis (C2), and partial loss of 3rd to 7th cervical vertebrae (C3-C7) (Fig 1D, upper box). In thoracic vertebrae (T2-T5), ossification centers were dislocated and spinous processes were fused (Fig 1D, lower box). The lumbar vertebrae were distorted (Fig 1F, asterisks) with dislocation of ossification centers and loss of pedicles (Fig 1F, arrowheads). Further, additional lumbar vertebrae were observed in most of *Oune/+* rats (Fig 1F, L7). These morphological abnormalities in *Oune/+* animals are summarized in Table 1.

**Table 1:** Statistics of skeletal phenotypes of new born animals from crosses between *Oune* heterozygote and F344/NSlc wild type.

	*Oune/+*	+/+
**Genotype** ^a^	** **	** **
Numbers	21	15
Ratio	0.583	0.416
**Skeletal abnormalities** ^**b**^ **, number (%)** ^**c**^		
Loss of atlas (C1) and axis (C2)	21 (100)	0 (0)
Fusion of spinous processes in T1-T5	21 (100)	0 (0)
Rib like bone fragments in L1	16 (76)	1 (7)
Additional lumber vertebra	18 (85)	0 (0)
Loss of sacral vertebrae	10 (47)	0 (0)
Additional sacral vertebra	2 (9)	0 (0)
Kinky tail	21 (100)	0 (0)

^a^ Animals with short kinky tails were counted as *Oune* heterozygotes.

^b^ The number of animals with abnormalities in a given skeletal structures is listed.

^c^ The percentage of animals with abnormalities is given in parentheses.

*Oune/+* females rarely became pregnant when bred with *Oune/+* males. The reason for this is unknown. It is probable that the vertebral anomalies in *Oune* rats influence sexual activity in this mating combination. Therefore, we obtained only a limited number of offspring from (*Oune/+* x *Oune/+*) mating pairs. No *Oune* homozygotes were observed among newborn animals, but were found within embryonic day of development (E) 15.5 embryos (Fig 1H and 1I). At this stage, they exhibited irregular positioning of the thoracic and lumbar vertebral bodies; two split columns were formed in homozygous embryos (Fig 1H and 1I, two zigzag lines). In addition, rib formation was attenuated in homozygotes. In contrast, linear placement of vertebral blocks was observed in wild type (Fig 1G, one line along the anterior-posterior axis).

**Fig 2:**
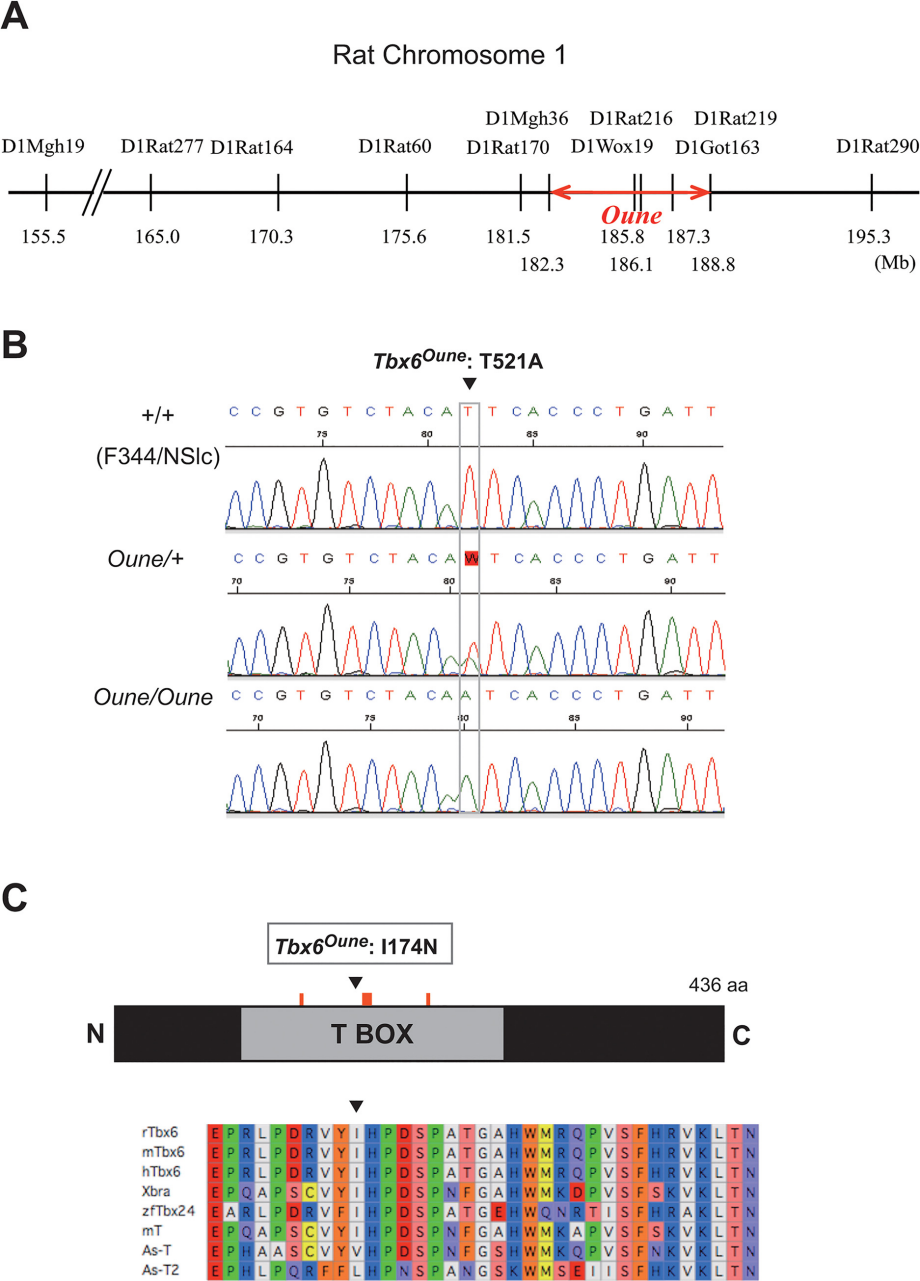
Positional candidate cloning of the causative gene for *Oune*. (A) Genetic mapping of the *Oune* locus indicates that the critical interval is located between D1Mgh36 and D1Got163 on rat chromosome 1. *Oune/+* rats, originally derived from the F344 strain, were crossed to the Bs/Ss strain, and overall 202 N2 animals were genotyped using 50 genome-wide microsattelite markers. (B) T to A transversion at the 521st base pair of *Tbx6* transcripts in *Oune* rats. The point mutation was identified from candidate sequencing of exons in the critical region. (C) The *Tbx6* ^*Oune*^ mutation causes an amino acid exchange in the T-box, the DNA binding domain, near a predictable dimerization interface (Up, arrowhead). Alignment of amino acid sequences among vertebrate and protochordate T-box genes (Down). The 174th isoleucine of rat *Tbx6* (arrowhead), which is exchanged to asparagine in *Oune*, is conserved among mouse, human, Xenopus, and zebrafish *Tbx6* and mouse *Brachyury* (mT). In ascidian T (As-T, As-T2) the isoleucine is changed to valine and leucine, but they are, like isoleucine, hydrophobic amino acids.

To identify the *Oune* mutation, genetic mapping and candidate sequencing were performed. For genetic mapping of the *Oune* locus, we used the Bs/SsNSlc strain (Japan SLC) for outcross. Overall 202 N2 backcross animals were genotyped using 50 genome-wide Simple Sequence Length Polymorphism (SSLP) markers listed in S1 Table. Pooling of DNA samples was used for the first screening (S1 Fig), and we found that the *Oune* locus was mapped to chromosome 1. Using an additional nine markers, the critical region was narrowed down to the approximately 6 Mb genomic region between *D1Mgh36* and *D1Rat219* (Fig 2A). Within the region, we selected *Tbx6* as a candidate, because it is known to play a crucial role in somite formation. Exonic DNA sequencing identified a T to A transition in the coding region of the *Tbx6* gene. The T521A transition in *Tbx6* was observed in both *Oune/+* and *Oune/Oune* genomic DNA, but not in wild type (Fig 2B). The *Oune* mutation causes a substitution from isoleucine to asparagine at amino acid 174 (I174N), which is located within the T-box domain near the predicted dimerization interface (Fig 2C, upper panel). Isoleucine 174 of rat Tbx6 is conserved across species (mouse, human, Xenopu*s*, and zebrafish) and in mouse *Brachyury* (T) (Fig 2C, lower panel). Although the isoleucine is exchanged to valine or leucine in ascidian T-box genes, they are, like isoleucine, hydrophobic amino acids. These results strongly indicate that *Oune* is an allele of *Tbx6*, and thus we renamed *Oune* as *Tbx6* ^*Oune*^.

**Fig 3:**
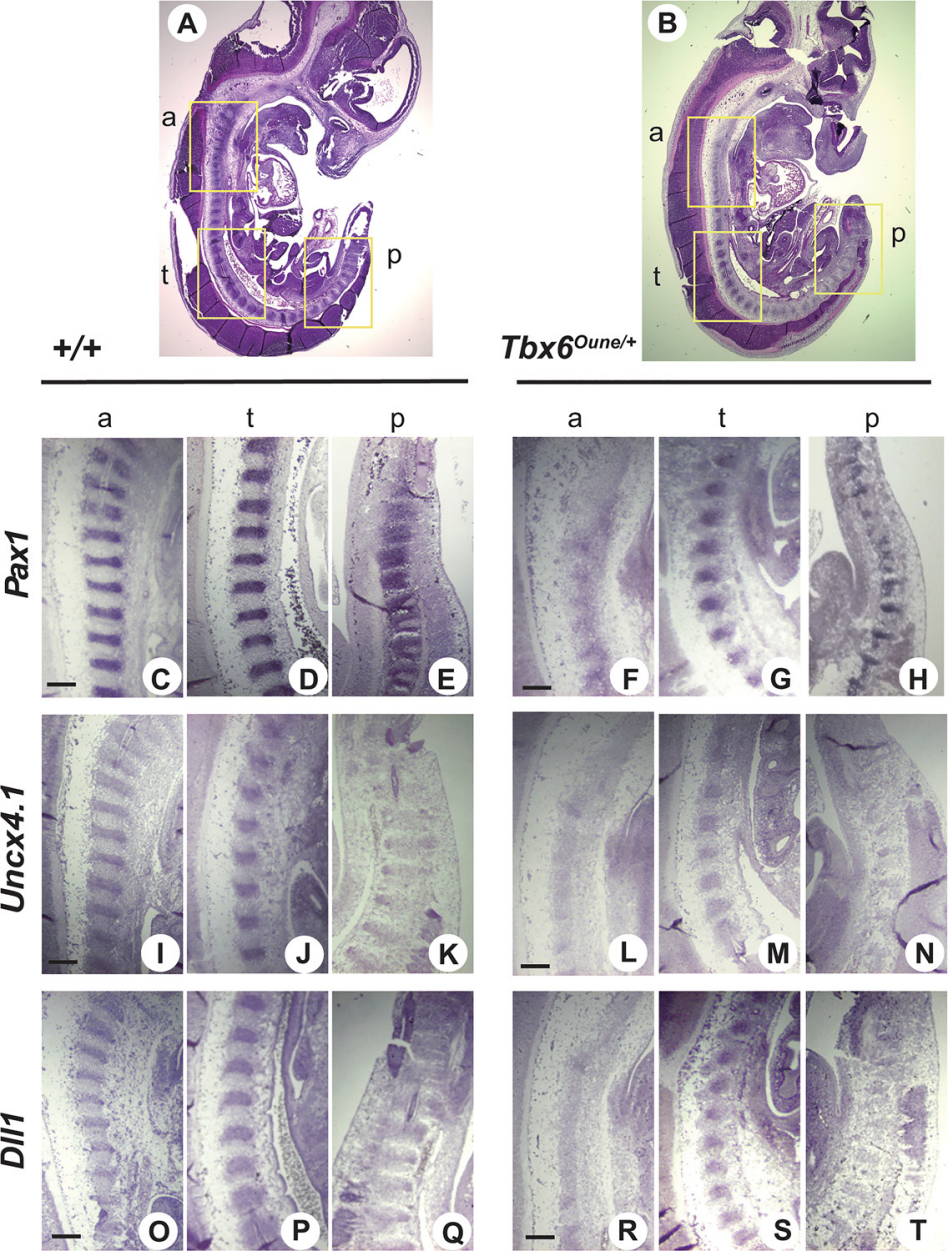
Somite pattering in *Oune/+* embryos. Sagittal sections of E14.5 +/+ and *Oune/+* embryos were used for hematoxylin and eosin staining (A, B) and *in situ* hybridization with various somite markers, *Pax1* (C-H), *Uncx4*.*1* (I-N), and *Dll1* (O-T). Incomplete somite patterning in the anterior region of *Oune/+* embryos was observed (B, box a). In the same embryo, somites in the trunk and posterior regions were morphologically normal (B, boxes t and p). In *Oune/+* embryos, *Pax1* expression was decreased in the anterior region (F), and somites were dislocated in the posterior region (H). Signals of markers for the caudal half of somites, *Uncx4*.*1* and *Dll1*, were reduced in the anterior and posterior regions of *Oune/+* embryos (L and N: *Uncx4*.*1*; R and T: *Dll1*). Scale bar, 200 μm.

**Fig 4:**
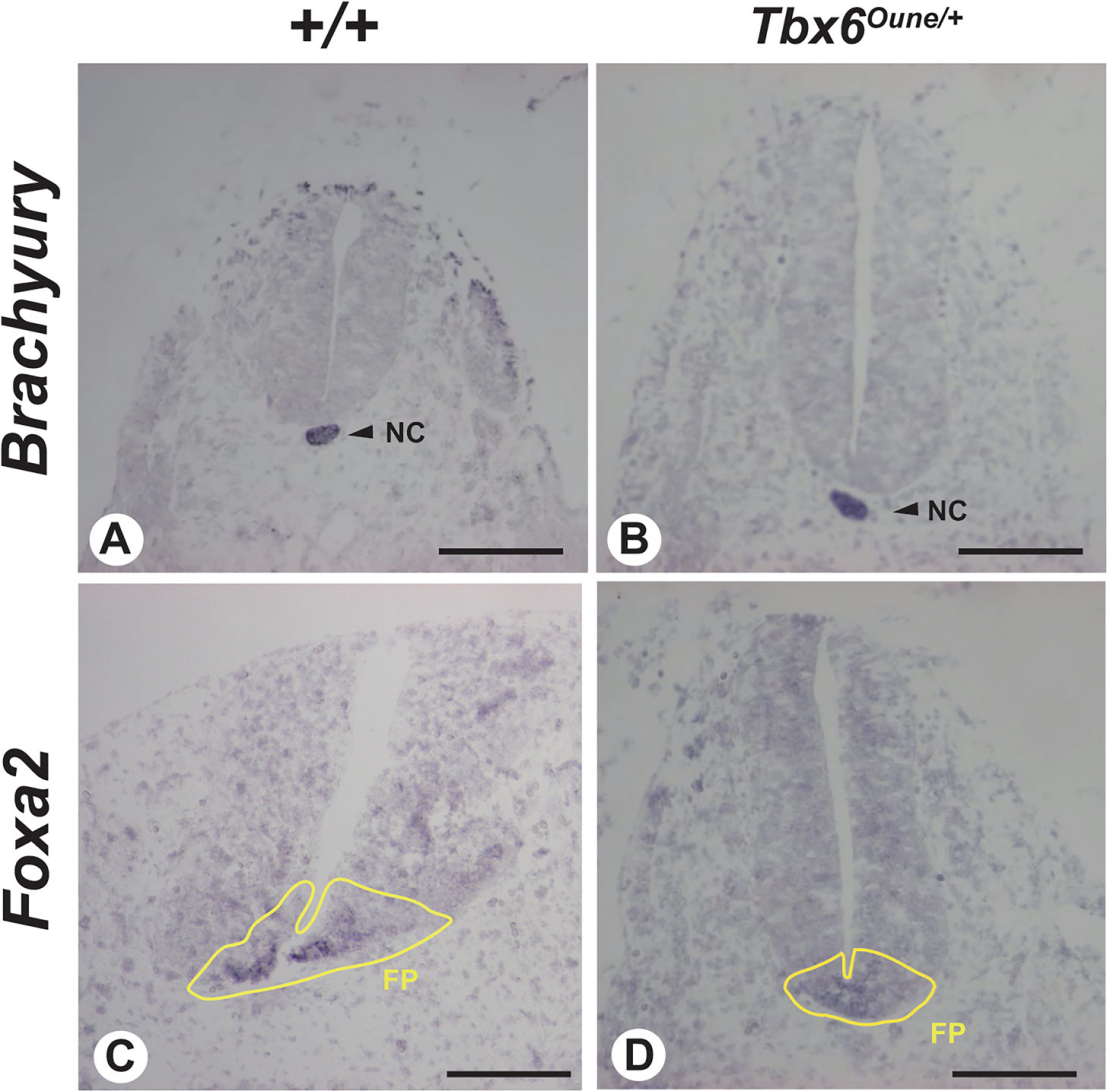
Notochord and floor plate patterning in *Oune/+* embryos. *In situ* hybridization analysis was performed on transverse sections through the trunk of E12.5 embryos using RNA probes for *Brachyury* (A, B), a notochord marker, and *Foxa2* (C, D), a floor plate marker. No expression change was observed between *+/+* and *Oune/+* embryos. Notochord (NC) is indicated by arrowheads and floor plate (FP) is enclosed by yellow lines. Scale bar, 200 μm.

**Fig 5:**
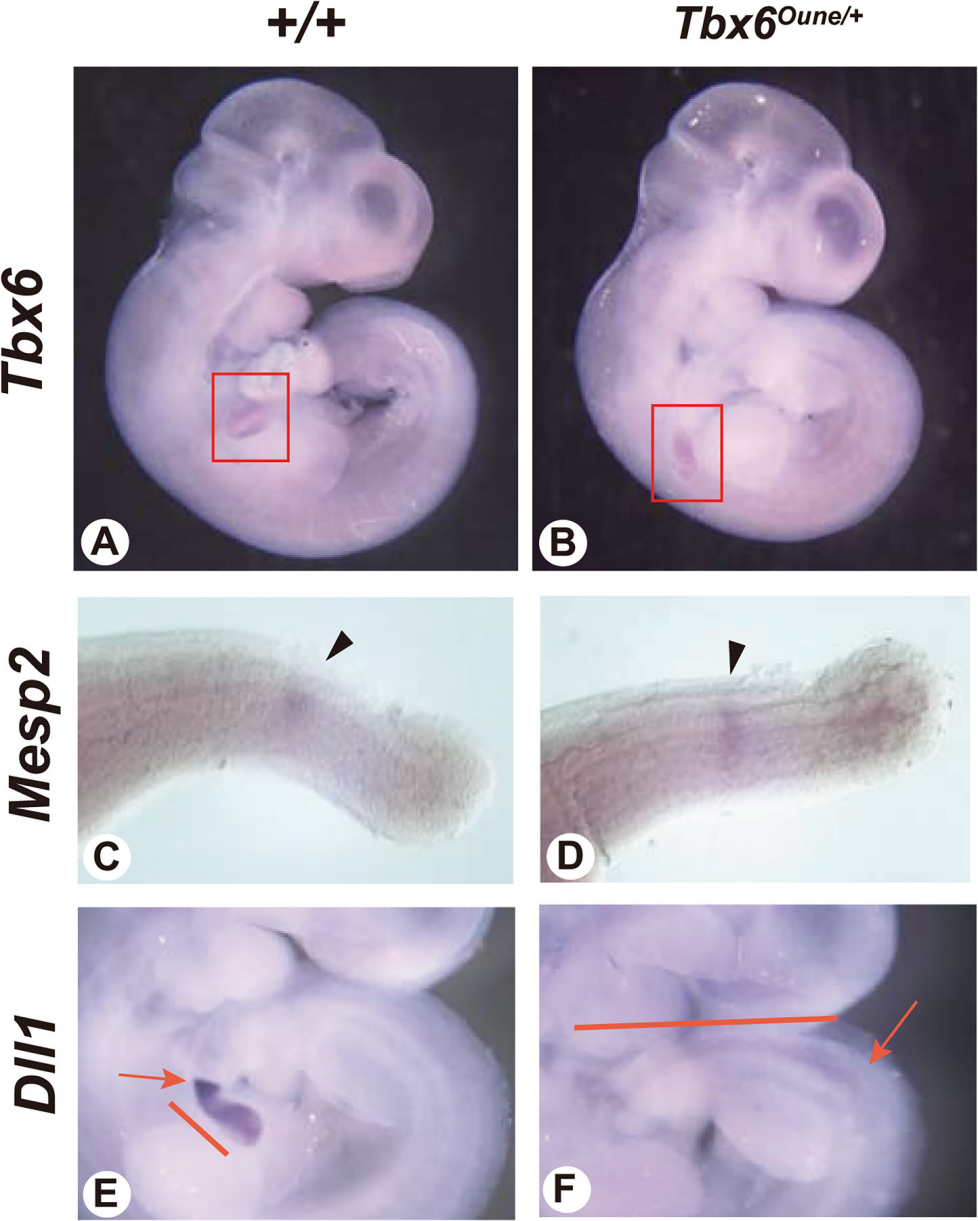
Altered expression of Notch pathway components in *Oune/+* embryos. Whole mount *in situ* hybridization with rat *Tbx6* (A, B), *Mesp2* (C, D), and *Dll1* (E, F) probes was performed using E12.5 +/+ and *Oune/+* embryos. Obvious changes of expression patterns and intensity were not observed. Red boxes (A, B) and arrowheads (C, D) indicate *Tbx6* expression in tail bud and *Mesp2* expression in presomitic mesoderm, respectively. The contiguous expression of *Dll1* in the presomitic mesoderm (bars, E, F) appear extended anteriorly in heterozygous mutant embryos. Arrows indicates expression borders between somites and presomitic mesoderm. Note that low levels of *Dll1* expression were observed in mutants. Original magnification: 20x (A, B); 90x (C, D); 40x (E-F).

We next analyzed pathogenesis of skeletal malformations in *Tbx6* ^*Oune/+*^ embryos obtained from (wild type x heterozygous) mating pairs. Hematoxylin eosin staining of E14.5 *Tbx6* ^*Oune/+*^ embryos showed that somites at the anterior and posterior ends were abnormally formed, and the borders between the somite compartments in these regions were obscure (Fig 3A, box a and p). In contrast, somites in the trunk region were formed without morphological abnormalities (Fig 3B, box t). Positions of the abnormal anterior and posterior somites are correlated with loss of cervical vertebrae and kinked tails of *Tbx6* ^*Oune/+*^ adults. *In situ* hybridization analyses on sections of E14.5 wild type and *Tbx6* ^*Oune/+*^ embryos were performed using various somite markers, *Pax1* (Fig 2C–2H), *Uncx4*.*1* (Fig 3I–3N), and *Dll1* (Fig 3O–3T). In *Tbx6* ^*Oune/+*^ embryos, expression of *Pax1*, a sclerotome marker, was attenuated in the anterior region (Fig 3F), but was normal in the trunk region (Fig 3G). In the posterior region, *Pax1* expression was also down regulated, and the regular positioning of the *Pax1* positive compartments was disturbed (Fig 3H). In addition, expression of *Uncx4*.*1* and *Dll1*, markers for the caudal half of somites, was also down regulated in the anterior and posterior regions of *Tbx6* ^*Oune/+*^ embryos (Fig 3I, 3N, 3R and 3T). No abnormality was detected in the trunk region (Fig 3M and 3S). Formation of the dorso-ventral axis of E12.5 *Tbx6* ^*Oune/+*^ embryos was normal judged by gene expression of a notochord marker, *Brachyury*, and a floor plate marker, *Foxa2* (Fig 4B and 4D). In addition, whole mount *in situ* hybridization using E12.5 embryos reveals that the *Oune* mutation did not affect expression of *Tbx6* itself in tail bud and *Mesp2* in presomitic mesoderm (Fig 5F and 5H). Nevertheless, the *Dll1* expression domain in presomitic mesoderm of *Tbx6* ^*Oune/+*^ embryos was expanded anteriorly (Fig 5F). Further, expression levels of *Dll1* in heterozygous embryos were reduced.

**Fig 6:**
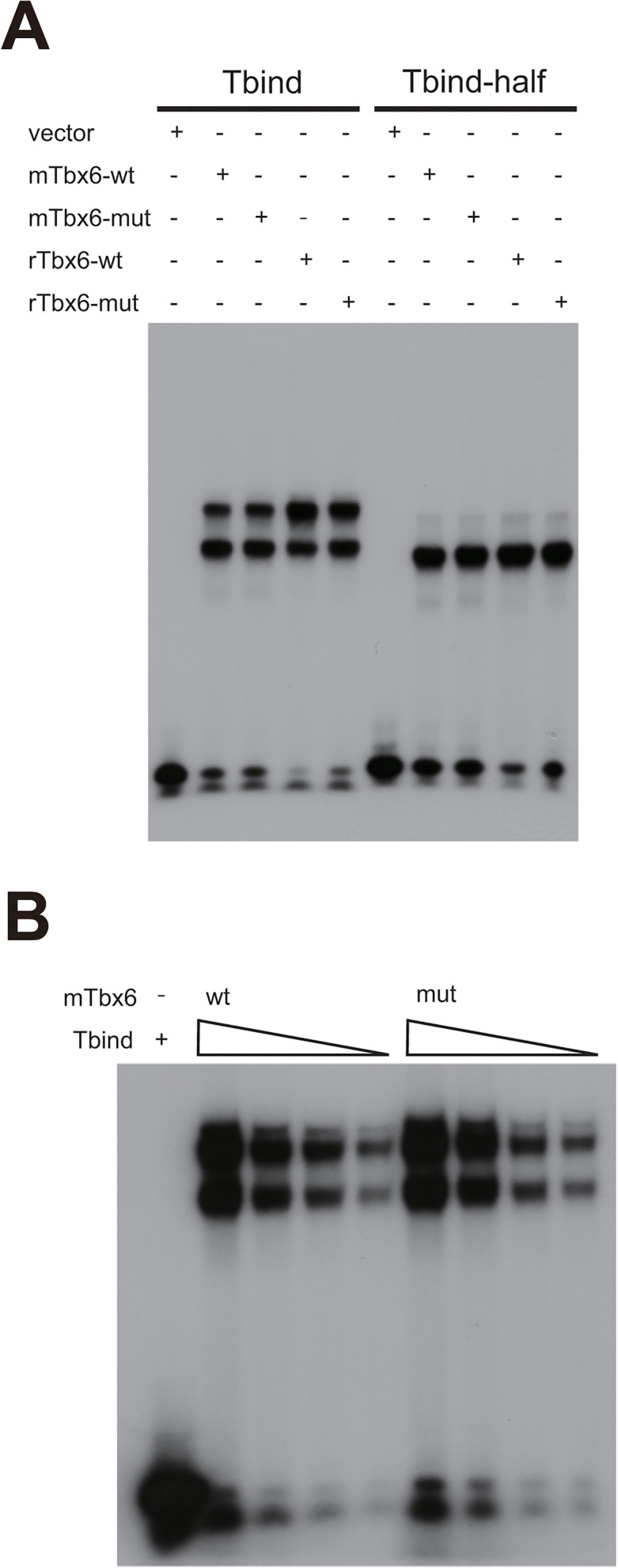
Electrophoretic mobility shift assay (EMSA) of *Tbx6* and *Tbx6* ^*Oune*^. (A) The *Tbx6* ^*Oune*^ allele did not influence the DNA binding ability of the T-box binding consensus sequences. *Tbx6* and *Tbx6* ^*Oune*^ (Tbx6 mut) showed no difference in binding ability to the Tbind probe, two T-box gene binding sites, and to the Tbind-half probe, a single binding site, judged from intensity of shifted bands. Free binding probes were shown at the bottom. (B) DNA binding ability of mutant Tbx6 was not changed. The Tbind probe concentration was diluted to one sixteenth, but no difference was detected between Tbx6-wt and mut. mTbx6 and rTbx6 represents mouse and rat Tbx6, respectively.

To search for functional differences between wild type Tbx6 and the I174N variant, Tbx6^Oune/+^, we performed various *in vitro* experiments. Initially, we thought that the *Oune* mutation might affect DNA binding activity of *Tbx6*, since the mutation is located within the T-box domain. Thus we employed electrophoretic mobility shift assay (EMSA) using double and single binding consensus sequence probes: Tbind and Tbindhalf, respectively. Tbind contains two T binding consensus motifs, CACAC [28] and AGGTGT [29], but Tbind-half contains only the AGGTGT motif. In EMSA with the Tbind probe, both mouse and rat Tbx6^Oun*e*^ and Tbx6 showed two shifted bands (Fig 6A). By contrast, Tbx6 with the Tbindhalf probe showed only one lower band (Fig 6A). In addition, serial dilutions of the Tbind probe did not show any changes in binding affinity of Tbx6^Oune^ (Fig 6B), and likewise serial dilutions of protein amount did affect affinity (data not shown). Thus, the I174N variant did not affect DNA binding activity of Tbx6.

**Fig 7:**
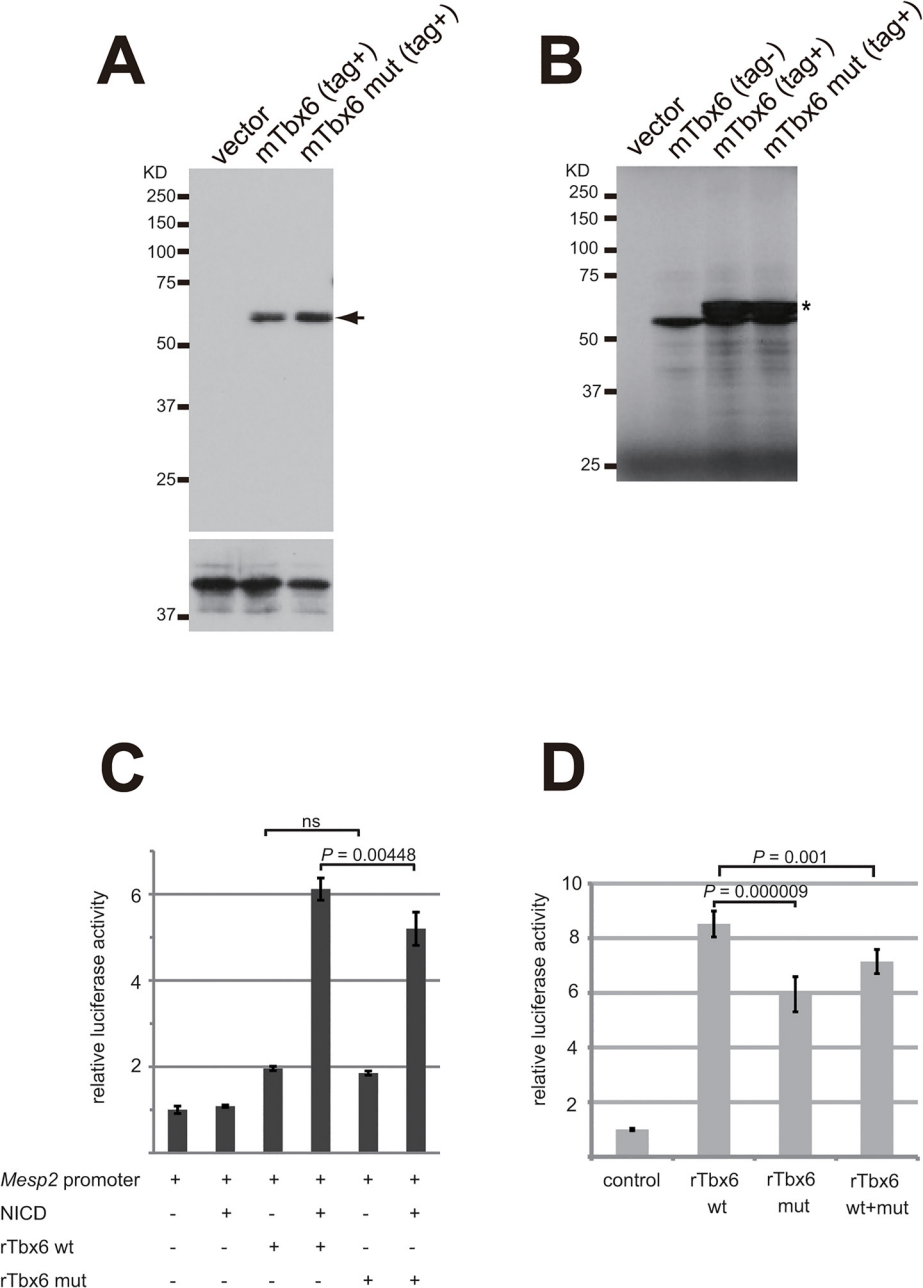
Translational efficiency and transcription activation ability of *Tbx6* proteins. (A) Western blotting analysis of S protein-tagged mTbx6 proteins. Cell lysate from transfectant of the *mTbx6* and *mTbx6* ^*Oune*^ expression constructs (Tbx6 and Tbx6 mut, respectively), in which the coding region of wild type and *Oune* mutant *mTbx6* are tagged with partial S protein sequences in N-terminus, was used with anti-S protein (the upper panel) and anti-USF2 (the lower panel) antibodies. Signals of S protein tagged mTbx6 are indicated by the arrow. (B) *In vitro* translation assays for mTbx6 and mTbx6 mut. Difference of translational efficiency between wild type and mutant mTbx6 was not observed. The S-tag mTbx6 constructs showed multiple translational initiations (tagged protein: asterisk). (C) Transcriptional activation properties of *Tbx6* and *Tbx6* ^*Oune*^ using a *Mesp2* promoter-luciferase reporter construct. *rTbx6* ^*Oune*^ activates transcription less effectively than *rTbx6* when a Notch intracellular domain (NICD) expression construct was cotransfected into C2C12 cultured cells. (D) Transcriptional activation properties of a mixture of *Tbx6* and *Tbx6* ^*Oune*^ constructs. Half-and-half of rTbx6 and rTbx6 mut constructs with NICD showed intermediate levels of luciferase activities. Assays were performed in triplicate. One-way analysis of variance was performed on data from all experiments, and significance was determined using Turkey's post hoc test. ns, not significant. mTbx6 and rTbx6 represents mouse and rat Tbx6, respectively.

We next thought that the *Oune* mutation might affect protein translational efficiency. Expression constructs for N-terminally S-protein-tagged (S-tag) *Tbx6* and *Tbx6* ^*Oune*^ were made accordingly and transfected into 293T cells. After 24 hours cell lysates were harvested, and western blotting was performed using anti-S-tag antibodies. Fig 6A shows no differences in the protein amount between Tbx6 and Tbx6^Oune^. In addition, *in vitro* translation/transcription experiments using rabbit reticulocyte lysates also indicated no difference in the efficiency of translation between Tbx6 and Tbx6^Oune^ (Fig 7B). Therefore, we assumed that the *Oune* mutation affect transcriptional activation ability of Tbx6. Thus we chose a *Mesp2* promoter-reporter construct to evaluate transcriptional activation properties of *Tbx6* ^*Oune*^, since it contains multiple *Tbx6* binding sites for transcriptional activation [26]. When transfected with the *Mesp2* reporter into mouse myoblast C2C12 cells, *Tbx6* and *Tbx6* ^*Oune*^ expression constructs showed similar luciferase activities (Fig 7C). It was reported that the Notch intracelluar domain (NICD) activates transcription of the *Mesp2* promoter in a *Tbx6*-dependent manner[26]. When cotransfected with an NICD expression construct, both *Tbx6* and *Tbx6* ^*Oune*^ showed greater luciferase activities. However, Tbx6^Oune^ activated *Mesp2* transcription significantly less efficiently than wild type Tbx6 (Fig 7C). Similar results were obtained with mouse *Tbx6* constructs (data not shown). Furthermore, a combined mixture of *Tbx6* and *Tbx6* ^*Oune*^ constructs showed an intermediated level of activation (Fig 7D).

## Discussion

The *Oune* locus was mapped to rat chromosome 1 by linkage analysis. In the critical region, we searched candidate genes that are expressed in early mesoderm and/or are functional in somite formation. We selected *Tbx6* for candidate sequencing because of its expression in presomitic mesoderm [30] and abnormal somitogenesis in *Tbx6* knockout mice [15] and *rib vertebra* (*rv*) mice, a hypomorphic allele of *Tbx6* [14,16]. We did not find any other candidates in the region according to the criteria. Further, no Notch pathway components, which play an important role in somitogenesis, were found in this region.

*Oune/+* rats exhibit similar skeletal abnormalities to *rv/rv* mice [13,31]. Both *Oune/+* and *rv/rv* mutants show morphological defects in cervical, lumbar, and sacral vertebrae. In *rv* mice, an insertion in the promoter region of the *Tbx6* locus causes low levels of *Tbx6* expression [14,16]. In the present study, we identified a missense mutation in the coding region of *Tbx6* in *Oune* rats. Because similar skeletal phenotypes are observed in *rv/rv* and *Oune/+* animals, *Oune* could be a hypomorphic allele of *Tbx6* as well as *rv*. Thus *Oune* was renamed as *Tbx6* ^*Oune*^.

Morphological phenotypes of *Tbx6* ^*Oune*^ rats are similar to that of other *Tbx6* mutants in vertebrates. In zebrafish, the *fused somites* (*fss*) mutant lacks somite borders along the antero-posterior axis, and the causative mutations were identified in *Tbx24* [32]. The zebrafish *Tbx24* gene recently has been identified as an ortholog of tetrapod *Tbx6* genes based on whole genome sequence data [33,34]. Thus, *Tbx6/Tbx24* is also important for somitogenesis in fish, indicating that its functions are well conserved between tetrapod and fish. In mammals, a murine null allele, *Tbx6* ^*tmPa1*^, was produced by genetic engineering [15]. *Tbx6* ^*tmPa1*^ homozygous embryos lack trunk somites and show enlarged tail buds and kinked neural tubes. They die by E12.5 because of vascular anomalies. In posterior paraxial tissues of *Tbx6* ^*tmPa1*^ homozygous embryos, neural tubes form instead of somites, resulting in formation of three neural tubes in the posterior part of the embryo. In contrast to the observed transdifferentiation of somites in *Tbx6* knockout mice, *Tbx6* ^*Oune/Oune*^ vertebral columns were formed but the vertebral blocks were positioned abnormally (Fig 1I and 1J). It is possible that in *Tbx6* ^*Oune/Oune*^ embryos somite abnormality is less severe than that of *Tbx6* knockout mice. Furthermore, *Tbx6* ^*Oune/Oune*^ embryos are still viable at E15.5 in rats, which corresponds to E14.5 in mice, without abnormal tail bud and vascular anomalies (data not shown), in contrast to the *Tbx6* knockout mice. Therefore, the phenotypes of the homozygous *Oune* allele are less severe than those of murine homozygous null allele, and as described above, morphological defects of heterozygous *Oune* animals are almost identical to those of the homozygous *rv* animals.

Recently, Sparrow *et al*. reported a stoploss mutation in *TBX6* detected by exome sequencing of Macedonian families with a dominant type of spondylocostal dysostosis [9]. The patients with the heterozygous *TBX6* mutation exhibited segmental congenital vertebral malformations including hemivertebrae and fused vertebral blocks, which resulted in short stature and scoliosis. These morphological defects in humans are similar to the skeletal phenotypes of *Tbx6* ^*Oune/+*^ rats (Fig 1C–1F). Interestingly, when the vertebral phenotype of heterozyous *Tbx6* knockout mice was re-examined, it was found that about half of the heterozygous mice had anomalies in cervical and sacral vertebrae in E14.5 *Tbx6* ^*tmPa1/+*^ embryos [9]. However, *Tbx6* ^*Oune/+*^ rats show additional anomalies in broad areas of the vertebral column, such as in thoracic, lumbar, and caudal vertebrae. Thus, heterozygosity for the *Oune* allele causes more severe vertebral phenotypes than does murine heterozygous null allele. Taking into account of the similar congenital vertebral malformations, we surmised that the vertebral anomalies of *Tbx6* ^*Oune/+*^ rats and spondylocostal dysostosis caused by the stoploss *TBX6* mutation would share a common molecular mechanism. However, the present study does not clarify whether modifier effects on the *Tbx6*-mediated pathways from different genomes or variations of *Tbx6* mutations themselves cause these phenotypic differences across species. Genome editing techniques [35,36] may enable us to elucidate different phenotypic effects of the same type of mutations in evolution.

The Notch pathway with *Mesp2* plays an important role in somite segmentation [2], and *Tbx6* participates in transcriptional regulation of *Mesp2* and *Dll1* by direct binding to their enhancers [25,37]. Thus, defects in *Tbx6* are expected to attenuate Notch signaling. In fact, in *Tbx6* ^*rv/rv*^ embryos, the expression pattern of Notch signaling components, such as *Dll1*, *Dll3* and *Notch1*, are altered [16,31]. In contrast, *Mesp2* expression in *Tbx6* ^*rv/rv*^ embryos is not altered[31]. Likewise, *Mesp2* expression in *Tbx6* ^*Oune/+*^ embryos was not changed (Fig 5D). Although the *Tbx6* ^*Oune*^ variant results in no change in DNA binding affinity to T-box consensus sequences, its transcriptional activity on a *Mesp2* enhancer construct was decreased when induced by the NICD (Fig 7C). Yasuhiko *et al*. showed that cotransfection of *Tbx6* with NICD dramatically increases reporter expression on a *Mesp2* enhancer [37]. Although this induction mechanism has not been well characterized, NICD may induce not yet identified proteins that bind to two neighboring *Tbx6* and activates transcription. It is probable that *Tbx6* ^*Oune*^ does not influence DNA binding activity itself but blocks protein-protein interaction. In addition, it has been reported that *Tbx6* directly binds to the mesoderm enhancer of a Notch component, *Dll1*, and activates its transcription [25]. Actually, whole mount *in situ* hybridization revealed that an anteriorly shifted change of *Dll1* expression was detected in both *Tbx6* ^*Oune/+*^ and *rv/rv* embryos (Fig 5F and [31]). Hence, *Tbx6* regulates *Mesp2* through NICD, but also regulates *Dll1* expression directly. Thus, the *Oune* mutation influences transcription activation only slightly; nevertheless, it suppresses the Notch pathways in somitogenesis via *Dll1*, NICD, and *Mesp2*.

The *Oune* mutation is located within the T-box domain, which is highly conserved among T-box family members. This domain is important for protein-protein interaction in addition to DNA binding activity. X-ray crystallography analysis showed that the T-box domain functionally includes a DNA binding domain and interfaces for dimer formation [29]. Although it is uncertain that Tbx6 forms as a dimer, Tbx6^Oune^ did not affect DNA binding activity to the oligonucleotide probes which contain a single and multiple binding consensus sequences (Fig 6). Among T-box family members, Tbx5 binds to Nkx2.5 with its T-box domain, and this binding is necessary for transcriptional activation [38]. Further, mouse and Xenopus Tbx6 interact with mouse Smad6 specifically, and the complex is degraded through ubiquitination [39]. If the *Oune* mutation influences Tbx6 binding affinity to Smad6, protein stability of Tbx6 could be dramatically changed. Thus, it remains possible that defects in protein-protein interactions of Tbx6^Oune^ causes the severe morphological abnormalities observed in *Oune* mutants.

In this study, a novel ENU-induced dominant mutation, *Oune*, in rats was isolated and characterized. The *Oune* mutation exhibits dominant vertebral malformations derived from defects in somite formation. Candidate positional cloning of *Oune* identified a missense mutation in *Tbx6*, and the mutation affected its transcriptional activity in a Notch dependent manner. It has been reported that a dominant type of familial spondylocostal dysostosis is caused by a mutation that affects the transcriptional activity of TBX6 with respect to Notch signaling. Therefore, further analyses of the molecular mechanisms leading to the morphological anomalies in *Oune* rats promise to provide insights into treatments and diagnosis of congenital spondylocostal dysostosis.

## Supporting Information

S1 Fig Pooling method used for genetic mapping of the *Oune* locus. Genome-wide scanning were performed using penal of 50 SSLP markers (S1 Table). Genomic DNA samples of (F344-*Oune* x BN) F1 x BN backcross (N2) rats were pooled, O: *Oune/+* (n = 15), C: +/+ (n = 15), B: BN/SsNSlc, F344/NSlc, M: DNA marker φX174-HaeIII digest. SSLP markers on chromosome 1, D1Mgh19 and D1Rat290, showed different band patterns of the PCR products between O and C, as shown in red box.
(EPS)

S1 Table SSLP markers used for genetic mapping of the *Oune* locus. (DOCX)

S2 Table SSLP markers used for fine mapping of the *Oune* locus on rat chromosome 1. (DOCX)

S3 Table Primers used for mutation screening of the *Tbx6* gene. (DOCX)

## References

[1] Christ B, Huang R, Scaal M (2004) Formation and differentiation of the avian sclerotome. Anat Embryol (Berl) 208: 333–350. 15309628 10.1007/s00429-004-0408-z

[2] Saga Y, Takeda H (2001) The making of the somite: molecular events in vertebrate segmentation. Nat Rev Genet 2: 835–845. 11715039 10.1038/35098552

[3] Turnpenny PD, Alman B, Cornier AS, Giampietro PF, Offiah A, Tassy O, et al (2007) Abnormal vertebral segmentation and the notch signaling pathway in man. Dev Dyn 236: 1456–1474. 17497699 10.1002/dvdy.21182

[4] Grünerberg H (1963) The Pathology of Development. Oxford: Blackwell.

[5] Sparrow DB, Chapman G, Dunwoodie SL (2011) The mouse notches up another success: understanding the causes of human vertebral malformation. Mamm Genome 22: 362–376. 10.1007/s00335-011-9335-5 21667129

[6] Offiah A, Alman B, Cornier AS, Giampietro PF, Tassy O, Wade A, et al (2010) Pilot assessment of a radiologic classification system for segmentation defects of the vertebrae. Am J Med Genet A 152A: 1357–1371. 10.1002/ajmg.a.33361 20503308

[7] Lander ES, Botstein D (1987) Homozygosity mapping: a way to map human recessive traits with the DNA of inbred children. Science 236: 1567–1570. 2884728 10.1126/science.2884728

[8] Rimoin DL, Fletcher BD, McKusick VA (1968) Spondylocostal dysplasia. A dominantly inherited form of short-trunked dwarfism. Am J Med 45: 948–953. 5722643 10.1016/0002-9343(68)90193-9

[9] Sparrow DB, McInerney-Leo A, Gucev ZS, Gardiner B, Marshall M, Leo PJ, et al (2013) Autosomal dominant spondylocostal dysostosis is caused by mutation in TBX6. Hum Mol Genet 22: 1625–1631. 10.1093/hmg/ddt012 23335591

[10] Naiche LA, Harrelson Z, Kelly RG, Papaioannou VE (2005) T-box genes in vertebrate development. Annu Rev Genet 39: 219–239. 16285859 10.1146/annurev.genet.39.073003.105925

[11] Herrmann BG, Labeit S, Poustka A, King TR, Lehrach H (1990) Cloning of the T gene required in mesoderm formation in the mouse. Nature 343: 617–622. 2154694 10.1038/343617a0

[12] Agulnik SI, Bollag RJ, Silver LM (1995) Conservation of the T-box gene family from Mus musculus to Caenorhabditis elegans. Genomics 25: 214–219. 7774921 10.1016/0888-7543(95)80128-9

[13] Nacke S, Schafer R, Habre de Angelis M, Mundlos S (2000) Mouse mutant "rib-vertebrae" (rv): a defect in somite polarity. Dev Dyn 219: 192–200. 11002339 10.1002/1097-0177(2000)9999:9999<::AID-DVDY1046>3.0.CO;2-9

[14] Watabe-Rudolph M, Schlautmann N, Papaioannou VE, Gossler A (2002) The mouse rib-vertebrae mutation is a hypomorphic Tbx6 allele. Mech Dev 119: 251–256. 12464437 10.1016/s0925-4773(02)00394-5

[15] Chapman DL, Papaioannou VE (1998) Three neural tubes in mouse embryos with mutations in the T-box gene Tbx6. Nature 391: 695–697. 9490412 10.1038/35624

[16] White PH, Farkas DR, McFadden EE, Chapman DL (2003) Defective somite patterning in mouse embryos with reduced levels of Tbx6. Development 130: 1681–1690. 12620991 10.1242/dev.00367

[17] Mashimo T, Yanagihara K, Tokuda S, Voigt B, Takizawa A, Nakajima R, et al (2008) An ENU-induced mutant archive for gene targeting in rats. Nat Genet 40: 514–515. 10.1038/ng0508-514 18443587

[18] Fuchs H, Lisse T, Abe K, Hrabé de Angelis M (2006) Screening for bone and cartilage phenotypes in mice; Hrabé de Angelis M, Chambon P, Brown S, editors. Weinheim: WILEY-VCH Verlag. 35–86 p.

[19] Abe K, Araki K, Tanigawa M, Semba K, Ando T, Sato M, et al (2012) A Cre knock-in mouse line on the Sickle tail locus induces recombination in the notochord and intervertebral disks. Genesis 50: 758–765. 10.1002/dvg.22035 22522943

[20] Taniguchi Y, Tanaka O, Sekiguchi M, Takekoshi S, Tsukamoto H, Kimura M, et al (2011) Enforced expression of the transcription factor HOXD3 under the control of the Wnt1 regulatory element modulates cell adhesion properties in the developing mouse neural tube. J Anat 219: 589–600. 10.1111/j.1469-7580.2011.01425.x 21929743 PMC3222838

[21] Neubuser A, Koseki H, Balling R (1995) Characterization and developmental expression of Pax9, a paired-box-containing gene related to Pax1. Dev Biol 170: 701–716. 7649395 10.1006/dbio.1995.1248

[22] Mansouri A, Voss AK, Thomas T, Yokota Y, Gruss P (2000) Uncx4.1 is required for the formation of the pedicles and proximal ribs and acts upstream of Pax9. Development 127: 2251–2258. 10804168 10.1242/dev.127.11.2251

[23] Bettenhausen B, Hrabe de Angelis M, Simon D, Guenet JL, Gossler A (1995) Transient and restricted expression during mouse embryogenesis of Dll1, a murine gene closely related to Drosophila Delta. Development 121: 2407–2418. 7671806 10.1242/dev.121.8.2407

[24] Kojima M, Takamatsu N, Ishii T, Kondo N, Shiba T (2000) HNF-4 plays a pivotal role in the liver-specific transcription of the chipmunk HP-25 gene. Eur J Biochem 267: 4635–4641. 10903495 10.1046/j.1432-1327.2000.01499.x

[25] White PH, Chapman DL (2005) Dll1 is a downstream target of Tbx6 in the paraxial mesoderm. Genesis 42: 193–202. 15986483 10.1002/gene.20140

[26] Yasuhiko Y, Haraguchi S, Kitajima S, Takahashi Y, Kanno J, Saga Y (2006) Tbx6-mediated Notch signaling controls somite-specific Mesp2 expression. Proc Natl Acad Sci U S A 103: 3651–3656. 16505380 10.1073/pnas.0508238103 PMC1450137

[27] Pui JC, Allman D, Xu L, DeRocco S, Karnell FG, Bakkour S, et al (1999) Notch1 expression in early lymphopoiesis influences B versus T lineage determination. Immunity 11: 299–308. 10514008 10.1016/s1074-7613(00)80105-3

[28] Conlon FL, Fairclough L, Price BM, Casey ES, Smith JC (2001) Determinants of T box protein specificity. Development 128: 3749–3758. 11585801 10.1242/dev.128.19.3749

[29] Muller CW, Herrmann BG (1997) Crystallographic structure of the T domain-DNA complex of the Brachyury transcription factor. Nature 389: 884–888. 9349824 10.1038/39929

[30] Chapman DL, Agulnik I, Hancock S, Silver LM, Papaioannou VE (1996) Tbx6, a mouse T-Box gene implicated in paraxial mesoderm formation at gastrulation. Dev Biol 180: 534–542. 8954725 10.1006/dbio.1996.0326

[31] Beckers J, Schlautmann N, Gossler A (2000) The mouse rib-vertebrae mutation disrupts anterior-posterior somite patterning and genetically interacts with a Delta1 null allele. Mech Dev 95: 35–46. 10906448 10.1016/s0925-4773(00)00323-3

[32] Nikaido M, Kawakami A, Sawada A, Furutani-Seiki M, Takeda H, Araki K (2002) Tbx24, encoding a T-box protein, is mutated in the zebrafish somite-segmentation mutant fused somites. Nat Genet 31: 195–199. 12021786 10.1038/ng899

[33] Ahn D, You KH, Kim CH (2012) Evolution of the tbx6/16 subfamily genes in vertebrates: insights from zebrafish. Mol Biol Evol 29: 3959–3983. 10.1093/molbev/mss199 22915831

[34] Windner SE, Bird NC, Patterson SE, Doris RA, Devoto SH (2012) Fss/Tbx6 is required for central dermomyotome cell fate in zebrafish. Biol Open 1: 806–814. 10.1242/bio.20121958 23213474 PMC3507223

[35] Hsu PD, Lander ES, Zhang F (2014) Development and applications of CRISPR-Cas9 for genome engineering. Cell 157: 1262–1278. 10.1016/j.cell.2014.05.010 24906146 PMC4343198

[36] Mashimo T (2014) Gene targeting technologies in rats: zinc finger nucleases, transcription activator-like effector nucleases, and clustered regularly interspaced short palindromic repeats. Dev Growth Differ 56: 46–52. 10.1111/dgd.12110 24372523

[37] Yasuhiko Y, Kitajima S, Takahashi Y, Oginuma M, Kagiwada H, Kanno J, et al (2008) Functional importance of evolutionally conserved Tbx6 binding sites in the presomitic mesoderm-specific enhancer of Mesp2. Development 135: 3511–3519. 10.1242/dev.027144 18849530

[38] Hiroi Y, Kudoh S, Monzen K, Ikeda Y, Yazaki Y, Nagai R, et al (2001) Tbx5 associates with Nkx2-5 and synergistically promotes cardiomyocyte differentiation. Nat Genet 28: 276–280. 11431700 10.1038/90123

[39] Chen YL, Liu B, Zhou ZN, Hu RY, Fei C, Xie ZH, et al (2009) Smad6 inhibits the transcriptional activity of Tbx6 by mediating its degradation. J Biol Chem 284: 23481–23490. 10.1074/jbc.M109.007864 19561075 PMC2749122

