# Correction: Novel ENU-Induced Mutation in *Tbx6* Causes Dominant Spondylocostal Dysostosis-Like Vertebral Malformations in the Rat

**DOI:** 10.1371/journal.pone.0134527

**Published:** 2015-07-29

**Authors:** Koichiro Abe, Nobuhiko Takamatsu, Kumiko Ishikawa, Toshiko Tsurumi, Sho Tanimoto, Yukina Sakurai, Thomas S. Lisse, Kenji Imai, Tadao Serikawa, Tomoji Mashimo

There is an error in the seventh author’s name. The middle initial was omitted. The correct name is: Thomas S. Lisse.
