# Supplementary figures and images for: Novel ENU-Induced Mutation in Tbx6 Causes Dominant Spondylocostal Dysostosis-Like Vertebral Malformations in the Rat
Source: PLoS One. 2015 Jun 19;10(6):e0130231. doi: 10.1371/journal.pone.0130231 (PMC4474719; doi:10.1371/journal.pone.0130231)

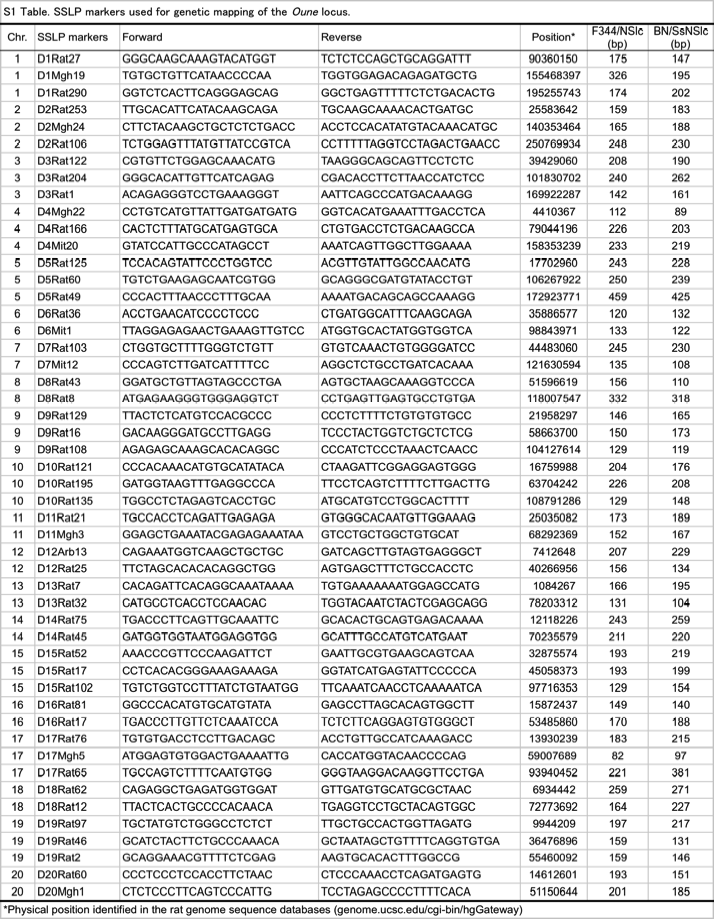

Supplement: S1 Table — (DOCX) [file pone.0130231.s002.docx]

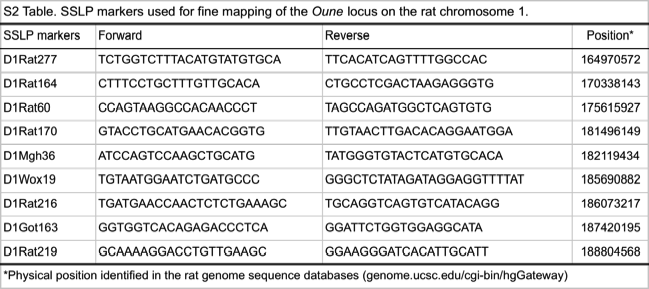

Supplement: S2 Table — (DOCX) [file pone.0130231.s003.docx]

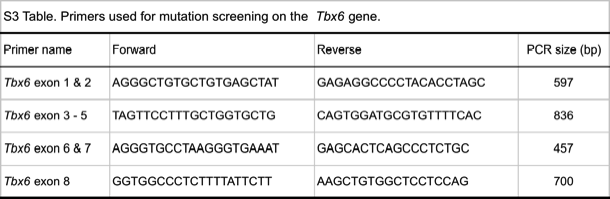

Supplement: S3 Table — (DOCX) [file pone.0130231.s004.docx]
